# Synthesis and characterization of gold nanoparticles from marine Micrococcus sp. OUS9

**DOI:** 10.6026/97320630016849

**Published:** 2020-11-30

**Authors:** Shanthi Kumari, Pabba Shivakrishna, K Sreenivasulu

**Affiliations:** 1 KLEF University, Guntur Andhra Pradesh, India; 2 Lavin laboratories, Hyderabad, India; 3 Osmania University, Department of microbiology, Hyderabad, India

**Keywords:** (EDX), (SEM), Micrococcus sp. OUS9, UV-Vis spectroscopy, Gold Nanoparticles

## Abstract

Studies on biological synthesis techniques of nanoparticles have been significantly expanded in recent years. This reduced adverse effects of chemical processing techniques. We describe the synthesis and characterization of gold nanoparticles from marine Micrococcus sp. OUS9 for potential application in nanobiotechnology.

## Background

Gold is one of the few elements of metal in the Earth's crust and occurs in aqueous solutions such as gold (0), gold (I) and gold (III) complexes. [1-2] AuNPs are uniquely described in terms of physical, chemical, electrical, electronic, magnetic, optical and biological properties as composed of bulk gold materials. [3,4] Based on their pronount biocompatibility, chemical inertness and physical properties, AuNPs have a good potential for biomedical applications including drug delivery.[5] Nanotechnology involves the processing of atomic-scale compounds, and nanoparticles (NPs) are materials with sizes of less than 100 nanometers (nm) [6]. While NPs have beneficial applications in human life, certain toxic effects can occur if they are absorbed into the body through the lungs, skin, open wounds and intestinal tract [7]. It is known that NPs are introduced into the atmosphere and animal bodies by effluents and disposals [8-9]. Hence, NPs can impose health risks, and assessing their nanotoxicity in vitro and in vivo is significant. Depending on the technique used to produce NPs, there are three types of NPs: physically, chemically and biologically produced NPs. Each production line has its own advantages and disadvantages [10]. Among the types of NP production techniques, the biological method is widely accepted because the use of living organisms in the production process is safer than other methods. In addition, various bacterial and fungal strains have the ability to produce NPs. The types of reductions vary depending on the nature of the active components responsible for the bio-reduction process. In other words, if microbial enzymes carry out the bio-reduction of the mediated toxic ion, then the reaction is enzymatic, and the active ingredients of microbial products are responsible for bio-reduction (i.e., polysaccharide or poly peptides), then non-enzymatic reactions are [11] responsible. Therefore, we describe the synthesis and characterization of gold nanoparticles from marine Micrococcus sp. OUS9 for potential application in nanobiotechnology.

## Methodology:

### Sampling site and sample collection:

Seawater and soil samples were collected from Nellore, Vishakapatnam and Bapatla. The water samples were collected in 500 ml sterile autoclavable collection bottles and the sediment samples were collected into sterile plastic polythene bags and sealed. The samples were collected under aseptic conditions and were placed on sterile icepacks until further process. These samples were inoculated within 1-2 h after collection [12].

### Isolation of marine bacteria:

All the samples were marked according to the locations collected, from each sample 60ul of water were spreaded over the Zobell's agar platespurchased from HI-Media, Mumbai, and incubated for 24 and 48 hrs at 28°C in incubator. After incubation the different colonies were transferred to the fresh sterile slants for further use [13].

### Screening for bioactive compounds producing bacteria using antagonism assay:

In vitro antagonism assay was carried out using a method developed elsewhere [14] against bacterial strains like Escherichia coli and Staphylococcus aureus [14]. The lawn culture was done by utilizing sterilized cotton swab and allowable to stay for 1 min. Ten micro liters of bacterial cultures was poured into wells and the petri dishes were incubated at 30°C overnight. Antagonistic interactions were scored based on the presence and appearance of inhibition zones. One of the isolated strains, which scored higher inhibition zone, was selected for further characterization.

### Molecular-based characterization:

By using 16s rRNA sequencing, the bacterial strain that showed the best inhibition against the selected pathogens was subjected to molecular identification [15] A phylogenetic tree was acquired with maximum probability demonstrating the evolutionary relationships between the chosen sequences.

### Extraction of crude extracts:

The fermentation was performed using 250ml capacity Erlenmeyer flasks for the selected active bacterial strains, containing 100ml of Zobell broth medium. The pure selected bacteria strain was inoculated with 1ml culture suspension for the sterilized fermented broth. On a rotary shaking incubator at 250 rpm, inoculated flasks were incubated at 28°C for five days. The fermented media was centrifuged for crude extract preparation at 10,000rpm or 20 min after incubation.

### Synthesis of AuNPs:

Micrococcus sp. OUS9 KLUF10 culture were centrifuged for 10 minutes to separate cells for 8000 rpm and cell free surnatants obtained were collected for the synthesis of AuNPs. The surnatant of the bacteria was mixed with 1 mM of Hydrogen tetrachloraurate (HAuCl4) which solution was heated in a microwave oven. Under the same laboratory conditions, the test tube with cell- free supernatant incubated. For further research, the tubes, which have witnessed ruby red formation, were confirmed for positive.

### Characterization:

AuNP synthesis was confirmed by the use of UV-visible spectroscopy by measuring the spectra from 400 to 700 nm. Functional groups were analyzed with a horizontal attenuated total reflectance for Synthesized AuNPs by FTIR. AuNPs were defined by SEM analysis in scale, shape and distribution. (XRD) Samples provided by adding the synthesized gold suspension to the 200 mesh carbon-coated copper panel, dried before SEM analysis.

### Antibacterial activity:

Anti-bacterial activity of AuNPs considered by the Agar technique of well diffusion was evaluated against various bacterial pathogens. Such as Salmonella sp (PM-08), staphylococcus aureus (PM-14), E. coli (PM-04), B subtitles, procured from PURE MICROBES, PUNE. The Plates were incubated at a temperature of 37°C in 18-24 hours, and the diameter of inhibition area (mm) were assessed at the end of the experiment and the activity index calculating was also calculated. The measurements took three distinct, set instructions and recorded average values.

## Results and Discussion:

In recent years, biomedical applications utilizing gold nanoparticles (GNPs) have been a very popular research area. [16-17] A broad variety of potential biomedical applications, i.e. drug delivery, (Mieszawska 2013 and Cho 2008) protein and pathogen identification, deoxyribonucleic acid labeling, fluorescent labeling, tissue engineering, and contrast agents for magnetic resonance imaging, have been explored. To improve the biocompatibility of GNPs it is preferable to use nontoxic reagents. All GNP-preparation methods are based on the reduction of gold ions, mostly as solutions of HAuCl4. Various reducing agents have been reported in the literature, the most common being sodium borohydride and sodium citrate [18].

**Figure 1:**
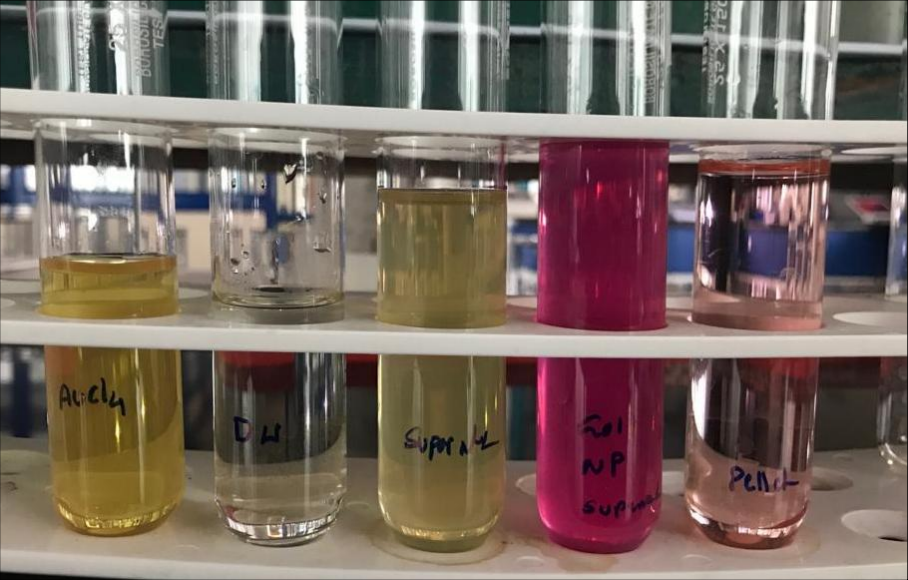
Synthesis of gold nanoparticles from Micrococcus sp. OUS9 supernatant.

In the present study, Micrococcus sp. OUS9 isolated from seawater was used for the synthesis of gold nanoparticles. Upon mixing the Micrococcus sp. OUS9 cell free supernatant with aqueous chloroauric acid, the solution transmuted color rapidly from pale yellow to vivid ruby-red, indicating the formation of AuNPs. The reduction of HAuCl4 was indicated by the colour changes of Micrococcus sp. OUS9 supernatant as shown in Figure 1. In the literature [19] it has been stated that AuNPs formation is detected by analyzing the colour shift of the reaction mixture. The bacteria can be an exceptional means for the extracellular synthesis of both gold nanoparticles.

**Figure 2:**
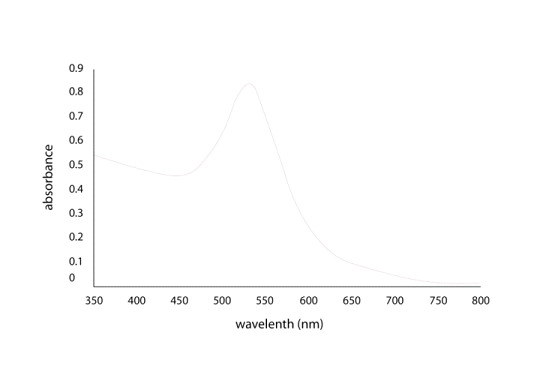
UV graph of gold nanoparticles from Micrococcus sp. OUS9 supernatant.

The UV-Vis absorption spectra of synthesized Au-Np from Micrococcus sp. OUS9 supernatant is shown clearly in Figure 2. The strong resonance peak at 540 nm was observed because of the gold nanoparticles 's surface plasm resonance (SPR). Due to collective resonance oscillations of valence electros, which interact with incoming electromagnetic radiation, ruby-red color of gold nanoparticles is observed.

**Figure 3:**
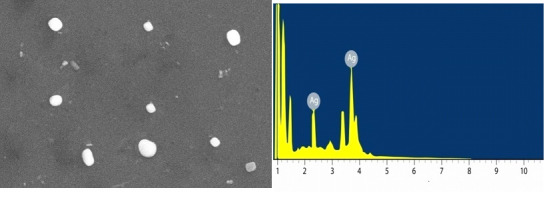
SEM and EDX of gold nanoparticles from Micrococcus sp. OUS9 supernatant.

Suspended centrifuged particles were collected for SEM analysis after a satisfactory synthesis process was completed. In this process, samples of gold nanoparticles were prepared by adding a drop of obtained suspension after centrifugation to the grids. The grids have been further dried and used for SEM research. Another advantage of TEM over SEM can be used to distinguish crystalline structures from amorphous structures using the selected area electron diffraction (SAED) technique [20,21]. The gold nanoparticles are shown like cubic in structure and moreover, the gold metal distribution beginners confirmed in our biogenic AuNPs by energy dispersive X-ray shown in the Figure 3.

**Figure 4:**
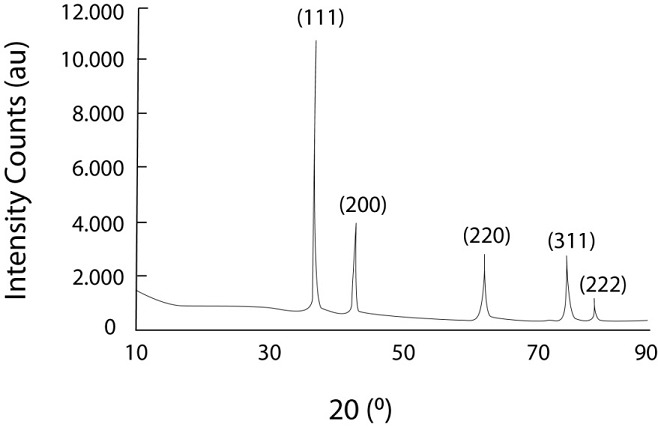
XRD of gold nanoparticles from Micrococcus sp. OUS9 supernatant.

The phase purity of the gold nanoparticles was confirmed by X-ray diffraction studies. Figure 4 shows the XRD overlay plots of gold nanoparticles prepared in inverse microemulsions using TritonX-100 as the surfactant at the different molar concentrations of aqueous HAuCl4 solution. All the diffraction patterns correspond to the monophasic nanocrystalline gold. The reflections belong to [111], [200], [220], [311] and [222] planes could be satisfactorily indexed to the pure crystalline metallic gold with face centred cubic structure.

**Figure 5:**
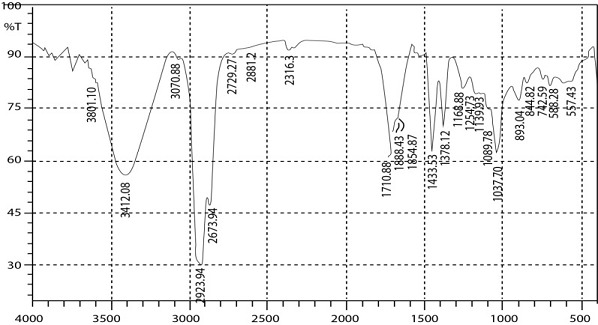
FTIR of gold nanoparticles from Micrococcus sp. OUS9 supernatant.

FTIR spectroscopic studies were carried out to investigate to find possible bioreducing agents present in the gold nanoparticles synthesized from Micrococcus sp. OUS9 supernatant (Figure 5). The interferrogram with a diameter of 3412 cm−1 is allocated to the N – H group of the peptide linkage present in the supernatant. The formation of C-C bonds is energetically preferred over S-C bonds, as the latter imposes extreme geometrical restrictions on the molecule more unique to the thiol group and less acidic relative to the alcohols, which makes the removal of hydrogen attached to the sulphur group. Concentration of amide relation in the supernatant is decreased Solution after the development of gold nanoparticles. Similar finding was in found in the study of 22 characterized the AuNPs produced by marine microalgal strain of Tetraselmis suesica. Table 1 shows the pathogenic bacteria and their zone of inhibition values in mm. Among the five test organisms selected for this study, maximal growth inhibition was observed in gold nanoparticles and which was almost equal to the results obtained using standard Streptomycin antibiotics. The AuNPs interacted with bacteria in all directions due to the multidimensional exposure of the NPs, which provided better interaction with microorganisms and enhanced antimicrobial activity.

## Conclusion

We describe the synthesis and characterization of gold nanoparticles from marine Micrococcus sp. OUS9 for potential application in nano biotechnology.
